# Effect of benzalkonium chloride–free travoprost on intraocular pressure and ocular surface symptoms in patients with glaucoma previously on latanoprost: an open-label study

**DOI:** 10.1186/s12886-015-0151-7

**Published:** 2015-11-12

**Authors:** Joao F. Lopes, Douglas A. Hubatsch, Patricia Amaris

**Affiliations:** Clinica Oftalmológica Pasteur, Luis Pasteur 5917 – Vitacura, Santiago, Chile; Alcon Laboratories, Inc., Fort Worth, TX USA; Clínica Oftalmológica del Caribe, Barranquilla, Colombia

**Keywords:** Benzalkonium chloride, Travoprost, Prostaglandin analog, Glaucoma, Safety, Efficacy

## Abstract

**Background:**

Prostaglandin analogs reduce intraocular pressure (IOP) in patients with open-angle glaucoma or ocular hypertension; however, these medications may affect the ocular surface and elicit ocular discomfort when preserved with benzalkonium chloride (BAK).

**Methods:**

This was an open-label, single-arm study conducted in Latin America from February 2012 to May 2013. Patients with open-angle glaucoma or ocular hypertension who were intolerant of latanoprost 0.005 % were transitioned to receive once-daily BAK-free travoprost 0.004 % containing polyquaternium-1 (Travatan® preserved with POLYQUAD® [PQ], Alcon Laboratories, Inc; Fort Worth, TX) for 12 weeks. Mean change in IOP from baseline (primary efficacy endpoint) and the percentage of patients who achieved a target IOP of ≤18 mmHg were evaluated at all on-therapy visits. Ocular hyperemia, patient preference, and self-projected adherence were assessed at week 12. Adverse events (AEs) were monitored throughout the study.

**Results:**

All enrolled patients were included in the analysis (*n* = 191); the majority of patients (90.6 %, *n* = 173/191) completed the study. Mean (SD) patient age was 67.5 (11.3) years, and mean baseline IOP was 14.8 mmHg. Mean IOP was reduced by 0.94 mmHg at week 6 and by 1.09 mmHg at week 12 (*P* < 0.001 for both). A greater percentage of patients achieved a target IOP of ≤18 mmHg at week 6 (93.1 %; *n* = 163/175) and week 12 (93.3 %; *n* = 166/178) compared with baseline (89.5 %; *n* = 171/191). There was a 10.5 % increase in the percentage of patients with “none/trace” amounts of hyperemia. Most patients preferred the study medication (81.5 %; *n* = 141/173) and were confident that they would adhere to their preferred medication (90.8 %; *n* = 157/173). No serious AEs were reported, and eye irritation (3.7 %; *n* = 7/191) was the most common treatment-related AE.

**Conclusions:**

Transitioning from BAK-containing latanoprost 0.005 % to BAK-free travoprost 0.004 % preserved with PQ reduced IOP in patients with open-angle glaucoma or ocular hypertension who were intolerant of latanoprost. BAK-free travoprost 0.004 % is a viable alternative for patients who require switching their IOP-lowering medications because of tolerability issues.

**Trial registration:**

ClinicalTrials.gov identifier, NCT01510145

## Background

Glaucoma is a progressive disorder characterized by optical neuropathy that may lead to blindness [1]. It is the second-leading cause of blindness worldwide, and in Latin America as many as 13.6 million people are estimated to be diagnosed with open-angle glaucoma by 2020 [2]. The disease may be especially burdensome in the Latin America region because diagnosis and treatment can be cost prohibitive [3]. Currently, the only evidence-based treatment paradigm for glaucoma focuses on reducing intraocular pressure (IOP) [1], which, if left untreated, has been associated with disease progression [4, 5].

The most commonly used IOP-reducing agents in Latin America are β-blockers, which reduce aqueous production [6], and prostaglandin analogs, which increase uveoscleral and trabecular meshwork aqueous outflow [7, 8]. Although both agents effectively reduce IOP, a greater reduction is typically obtained with prostaglandin analogs [9]. In addition, β-blockers have been associated with systemic cardiac and respiratory adverse effects, which may limit their use in some patients [10–14]. All prostaglandin analogs (eg, bimatoprost, latanoprost, and travoprost) have a similar IOP-lowering efficacy (8.0 to 8.7 mmHg from baseline) [15] of approximately 30 %, but they differ in their incidences of hyperemia [16].

In a long-term study of patients with primary open-angle glaucoma, travoprost 0.004 % preserved with benzalkonium chloride (BAK) significantly decreased mean 24-h IOP from 23.4 mmHg at baseline to approximately 16.8 mmHg through 5 years of treatment; mean IOP was reduced by approximately 28 % [17]. To improve tolerability, a BAK–free formulation of travoprost 0.004 % containing a polyquaternium-1 preservative has been developed. BAK is a quaternary ammonium compound preservative [18] that has been associated with a variety of adverse ocular symptoms (eg, burning/stinging, hyperemia, foreign body sensation, reduced tear production) [19–21] and detrimental effects on corneal epithelium cell function [22–25]. POLYQUAD® (PQ) is a BAK alternative used predominately in contact lens solutions and artificial tears [23] and has been shown to elicit fewer cytotoxic effects than BAK in vitro [23, 24]. Clinically, PQ-preserved ophthalmic solutions appear to reduce ocular discomfort associated with drop administration without affecting efficacy [26, 27]. For example, PQ-preserved travoprost 0.004 % was associated with a slightly reduced incidence of eye irritation compared with travoprost 0.004 % containing BAK while providing similar reductions in IOP [26]. However, the benefit of switching patients who are intolerant of BAK-preserved prostaglandin analogs such as latanoprost to BAK-free formulations containing PQ has not been thoroughly evaluated.

The purpose of the present study was to assess the efficacy and tolerability of transitioning from BAK-containing latanoprost 0.005 % to BAK-free travoprost 0.004 % containing PQ in patients with open-angle glaucoma or ocular hypertension.

## Methods

### Study design and treatment

This 12-week, multicenter, open-label, single-arm study (NCT01510145) was conducted in Argentina, Chile, and Colombia from February 2012 to May 2013. Patients with open-angle glaucoma or ocular hypertension who, in the opinion of the investigator, would benefit from discontinuation of latanoprost 0.005 % ophthalmic solution because of tolerability issues were transitioned to receive BAK-free travoprost 0.004 % (Travatan® preserved with PQ; Alcon Laboratories, Inc., Fort Worth, TX) once daily at approximately 8 pm for 12 weeks. The study protocol was reviewed and approved by the following independent review boards: Comité Independiente de Ética para Ensayos en Farmacología Clinica (Buenos Aires, Argentina), Comité Ético Científico del Servicio de Salud Metropolitano Oriente (Santiago, Chile), Comité Ético de la Fundación Oftalmológica Los Andes (Santiago, Chile), Comité de Ética del Servicio del Salud Metripolitano Sur Oriente (Santiago, Chile), Comité de Etica en Investigación del Hospital Clínico UC (Región Metropolitana, Chile), Comité de Revisión de Estudios de Investigación (Medellin, Colombia), and Clínica Oftalmológica del Caribe (Barranquilla, Colombia). The study was performed in accordance with ICH Good Clinical Practice guidelines. All patients provided written informed consent before initiation of study procedures.

### Patients

Adult patients were allowed to participate if they were diagnosed with ocular hypertension or open-angle glaucoma in at least 1 eye, had been receiving latanoprost 0.005 % ophthalmic solution monotherapy (including BAK-preserved generics) for ≥4 weeks before the screening visit, and would benefit from transitioning to BAK-free travoprost. IOP must have been <30 mmHg in both eyes while receiving latanoprost and had to pose no threat to vision stability or the optic nerve. Best corrected visual acuity was required to be >20/200 Snellen (1.0 logMAR) in both eyes.

Women who were pregnant or lactating were not allowed to participate. Patients were also excluded if they had a history of allergy, hypersensitivity, or poor tolerance to components of BAK-free travoprost containing PQ; had any abnormality that precluded reliable applanation tonometry; had corneal dystrophies, concurrent infectious or noninfectious conjunctivitis, keratitis, uveitis, dry eye, keratoconjunctivitis sicca, or progressive retinal or optic nerve disease from any cause; had a history of or were at risk for uveitis or cystoid macular edema; or had conventional or laser surgery in either eye ≤3 months before screening. Patients who were receiving systemic medications that could affect IOP (eg, oral β-adrenergic blockers, α-agonists and blockers, angiotensin-converting enzyme inhibitors, and calcium channel blockers) must have been on a stable dosage for ≥7 days before screening.

### Outcomes

Intraocular pressure was measured using Goldmann applanation tonometry at screening and baseline and at on-therapy visits at weeks 6 and 12; all on-therapy measurements were obtained at approximately the same time of day (±1 h) as baseline measurements. Change in IOP from baseline to week 12 was the primary efficacy endpoint; the percentage of patients who achieved a target IOP of ≤18 mmHg was also evaluated. Ocular hyperemia was scored as 0 (“no hyperemia”) to 3 (“severe hyperemia”) at baseline and week 12. Also at week 12, patients self-reported ocular discomfort on a scale ranging from 0 (“no discomfort”) to 9 (“substantial discomfort”). At the end of the study, patients were asked to identify which medication they preferred: latanoprost 0.005 % or BAK-free travoprost 0.004 %. Based on their medication preference, patients chose their level of confidence (ie, “not at all confident,” “somewhat confident,” or “very confident”) in answer to the question, “How confident are you that you will use your glaucoma medication as prescribed, if your doctor prescribed (a) your preferred medication, (b) your nonpreferred medication, (c) medication that caused burning or stinging, and (d) medication that did not cause burning or stinging?” Adverse events (AEs) were collected at each study visit and coded based on the Medical Dictionary for Regulatory Activities (version 15.0).

### Statistical analyses

The change in IOP from baseline to week 12 was analyzed using a mixed model including visit as a fixed effect and patient as a random effect. *P* values of <0.05 were considered statistically significant.

## Results

### Patients

A total of 191 patients were enrolled, received study medication, and were included in the safety and full-analysis datasets (ie, patients who received ≥1 dose of study medication); 173 (90.6 %) patients completed the study. Reasons for study discontinuation were AEs (*n* = 6), personal reasons (*n* = 6), loss to follow-up (*n* = 5), or other reasons (*n* = 1). At baseline, patients had a mean (range) age of 67.5 (23–89) years and were mostly white (78.0 %; *n* = 149/191) and female (72.8 %; *n* = 139/191).

### Efficacy

**Fig. 1:**
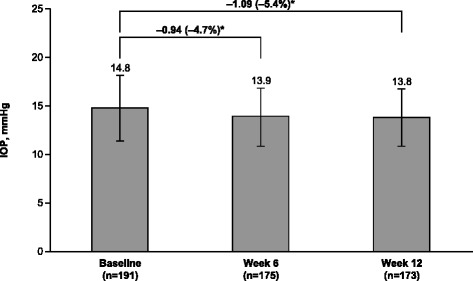
Intraocular pressure throughout the study. IOP = intraocular pressure. *P < 0.001

**Fig. 2:**
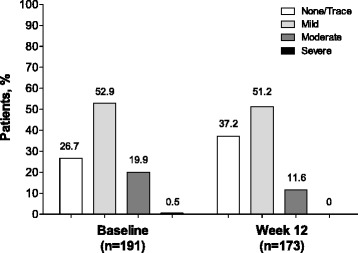
Ocular hyperemia severity at baseline and week 12. Hyperemia was assessed by study personnel using a scoring system ranging from 0 (no hyperemia) to 3 (severe hyperemia)

**Fig. 3:**
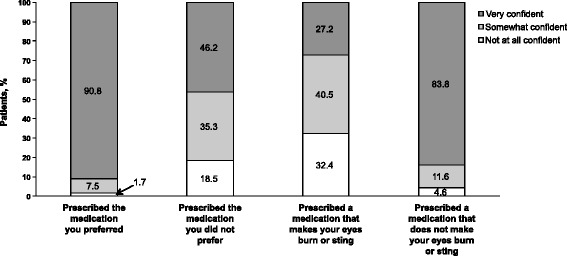
Responses to the adherence question by category. Patients chose their level of confidence (ie, “not at all confident,” “somewhat confident,” or “very confident”) in answer to the question, “How confident are you that you will use your glaucoma medication as prescribed, if your doctor prescribed the medication?”

Mean IOP at baseline was 14.8 mmHg. Mean IOP decreased from baseline to week 12 by 1.09 mmHg (5.4 %; *P* < 0.001; Fig. 1); a similar reduction in IOP was observed at week 6 (0.94 mmHg; 4.7 %; *P* < 0.001). The percentage of patients achieving the target IOP of ≤18 mmHg increased from 89.5 % (*n* = 171/191) at baseline to 93.1 % (*n* = 163/175) at week 6 and 93.3 % (*n* = 166/178) at week 12. Mean ocular hyperemia score decreased from 0.94 at baseline to 0.74 at week 12. The percentage of patients with “none/trace” hyperemia increased from 26.7 % (*n* = 51/191) at baseline to 37.2 % (*n* = 64/172) at study end (10.5 % increase; Fig. 2). Mean (SD) self-assessed ocular discomfort score was 1.83 (2.33), which was on the low end of the scale, and indicates minimal discomfort levels. More patients preferred the study medication over their prior medication (81.5 % [*n* = 141/173] vs 18.5 % [*n* = 32/173]) when given the choice between them. Most patients were very confident that they would use their preferred medication as prescribed (preferred medication: 90.8 %, *n* = 157/173; nonpreferred medication: 46.2 %, *n* = 80/173) and indicated a high adherence level for medications free of local irritation effects (83.8 %, *n* = 145/173; Fig. 3).

### Safety

Eight patients discontinued BAK-free travoprost because of 9 AEs (ocular hyperemia, *n* = 3; eye irritation, *n* = 2; conjunctival edema, *n* = 1; foreign body sensation in eyes, *n* = 1; pingueculitis, *n* = 1; headache, *n* = 1). No serious AEs were reported. Forty-two AEs were reported by 29 patients (15.2 %; *n* = 29/191) during the study. The most commonly reported AEs were eye irritation (3.7 %; *n* = 7/191), eye pruritus (3.1 %; *n* = 6/191), and eye pain (2.6 %; *n* = 5/191). Most AEs (83.3 %; *n* = 35/42) were considered mild and related to treatment (Table 1). AEs severe in intensity were reported in 4 patients and included ocular hyperemia (*n* = 3), eye irritation (*n* = 1), and conjunctival edema (*n* = 1).

**Table 1:** Treatment-related adverse events

AE, n (%)	BAK-Free Travoprost
	(*n* = 191)
Eye irritation	7 (3.7)
Eye pruritus	6 (3.1)
Eye pain	5 (2.6)
Foreign body sensation in eyes	3 (1.6)
Ocular hyperemia	3 (1.6)
Abnormal sensation in the eye	2 (1.0)
Blurred vision	2 (1.0)
Headache	2 (1.0)
Photophobia	1 (0.5)
Pingueculitis	1 (0.5)
Skin hyperpigmentation	1 (0.5)
Conjunctival edema	1 (0.5)
Dry eye	1 (0.5)

*AE* = adverse event; *BAK* = benzalkonium chloride

## Discussion

In this study, patients who transitioned because of intolerability from BAK-containing latanoprost to BAK-free travoprost preserved with PQ had a reduction in IOP after the switch. Additionally, patients reported fewer ocular surface abnormalities and less hyperemia after transitioning to the BAK-free medication. Few AEs were reported and most were mild in severity.

Previous studies have demonstrated an approximately 1 mmHg reduction in IOP after transitioning from BAK-containing latanoprost to BAK-free travoprost preserved with sofZia® (Alcon Laboratories, Inc.) [28, 29]. In the present study, a similar reduction of 1.09 mmHg in IOP was observed after transitioning from BAK-containing latanoprost to BAK-free travoprost preserved with PQ. The reduction in IOP was observed by week 6 and was maintained throughout the study, enabling more patients to reach the target IOP of ≤18 mmHg.

Ocular surface disease is prevalent among patients with glaucoma, especially those who receive treatment with ophthalmic solutions [30]. Although the exact mechanism for this remains unknown, BAK has been associated with a variety of ocular symptoms such as burning, stinging, foreign body sensation, and dry eye [19, 20, 30]. BAK is a detergent used in ophthalmic solutions to prevent microbial growth and perpetuate medication distribution into the eye [18]. However, it also disrupts cellular processes [18, 25] and reduces tear film production [20] and stability [31, 32]. Eliminating or reducing exposure to BAK in ophthalmic solutions improves tear film stability [20], reduces conjunctival hyperemia [21], and improves dry eye symptoms [30, 33]. For example, transitioning from BAK-containing latanoprost to BAK-free travoprost preserved with sofZia® significantly decreased hyperemia severity and superficial punctate keratitis [28]. In the current study, transitioning from BAK-containing latanoprost to BAK-free travoprost significantly reduced the incidence and severity of hyperemia. The percentage of patients with mild hyperemia was relatively unchanged after transitioning to BAK-free travoprost; however, the percentage of patients who reported moderate or severe hyperemia decreased after the transition and the percentage of patients with no hyperemia or only trace hyperemia increased, suggesting there was a net shift toward less severe hyperemia.

Glaucoma is a progressive disorder that requires chronic daily medication; however, up to 70 % of patients are nonadherent to their medications within the first year [34]. Hyperemia, pain, burning, and ocular discomfort are common causes of patient nonadherence. In the current study, most patients preferred BAK-free travoprost with PQ over latanoprost containing BAK. This preference may be related to the low ocular discomfort reported with BAK-free travoprost and the reduction in hyperemia severity. Indeed, the percentage of patients who preferred BAK-free travoprost (81.5 %) was similar to the percentage of patients who were very confident that they would adhere to a treatment regimen that did not cause burning or stinging (83.8 %). Patients reported high anticipated adherence for their preferred treatment and for a treatment that did not make their eyes burn or sting; a potential increase in adherence with BAK-free travoprost compared with BAK-preserved latanoprost may have contributed to the increased number of patients who achieved the IOP goal of ≤18 mmHg at week 12 compared with baseline.

Interpretation of the current results is limited by the design of the study. Some of the reductions in ocular hyperemia severity may be attributable to observer bias, given the open-label nature of the study. Further, the proportion of patients receiving generic or branded latanoprost at screening was not assessed; potential differences in excipients of these formulations may have differentially influenced baseline severity of ocular hyperemia. The suggestive nature of the questions assessing confidence in treatment adherence with regard to medication preference and burning or stinging may have introduced bias in patient responses. Transitioning all patients to BAK-free travoprost prevented comparison with other prostaglandin analogs and did not allow comparison with remaining on latanoprost; however, this study was specifically designed to evaluate therapeutic transition among patients for which intolerability necessitated a therapeutic change. The 12-week study duration did not allow conclusions regarding the long-term tolerability and efficacy of BAK-free travoprost with PQ.

## Conclusions

Transitioning patients who were intolerant of latanoprost 0.005 % to BAK-free travoprost 0.004 % reduced IOP, improved hyperemia levels, and demonstrated few side effects. Most patients preferred BAK-free travoprost over latanoprost and rated themselves more likely to be compliant with treatment.

## Abbreviations

AE: Adverse events
BAK: Benzalkonium chloride
ICH: International Conference on Harmonisation
IOP: Intraocular pressure
PQ: POLYQUAD® (Polyquaternium-1)
SD: Standard deviation
